# ACSL4-Mediated Ferroptosis and Its Potential Role in Central Nervous System Diseases and Injuries

**DOI:** 10.3390/ijms241210021

**Published:** 2023-06-12

**Authors:** Bowen Jia, Jing Li, Yiting Song, Chengliang Luo

**Affiliations:** Department of Forensic Medicine, School of Basic Medicine and Biological Sciences, Soochow University, Suzhou 215123, China

**Keywords:** ACSL4, ferroptosis, nervous system diseases, nervous system injuries

## Abstract

As an iron-dependent regulated form of cell death, ferroptosis is characterized by iron-dependent lipid peroxidation and has been implicated in the occurrence and development of various diseases, including nervous system diseases and injuries. Ferroptosis has become a potential target for intervention in these diseases or injuries in relevant preclinical models. As a member of the Acyl-CoA synthetase long-chain family (ACSLs) that can convert saturated and unsaturated fatty acids, Acyl—CoA synthetase long-chain familymember4 (ACSL4) is involved in the regulation of arachidonic acid and eicosapentaenoic acid, thus leading to ferroptosis. The underlying molecular mechanisms of ACSL4-mediated ferroptosis will promote additional treatment strategies for these diseases or injury conditions. Our review article provides a current view of ACSL4-mediated ferroptosis, mainly including the structure and function of ACSL4, as well as the role of ACSL4 in ferroptosis. We also summarize the latest research progress of ACSL4-mediated ferroptosis in central nervous system injuries and diseases, further proving that ACSL4-medicated ferroptosis is an important target for intervention in these diseases or injuries.

## 1. Introduction

Cell death is a common process in all living organisms, and diverse types of cell death have been classified over time. According to the latest recommendations of the Cell Death Nomenclature Committee in 2018, there are two types of cell death, that is, accidental cell death (ACD) and regulated cell death (RCD) [1]. As a new form of regulated cell death (RCD), ferroptosis is iron-dependent and characterized by the intracellular accumulation of lipid peroxides to lethal levels [2]. However, ferroptosis is distinct from apoptosis, various forms of necrosis, and autophagy in terms of morphology, biochemistry, and gene expression. The typical symptoms of mitochondrial cristae reduction or disappearance and outer membrane rupture differ from those of apoptotic cells, which are characterized by membrane blistering and contraction [3]. ACSLs are a family of enzymes that can convert saturated and unsaturated fatty acids with chain lengths of 8–22 to fatty acid acyl-CoA esters [4]. ACSLs mediate fatty acid metabolism and are widely involved in endoplasmic reticulum ER stress and ferroptosis. In particular, ACSL4 is a key enzyme in the production of lipid peroxides, thus promoting ferroptosis in cells [5]. In this review, we will mainly discuss the role of ACSL4 in the process of ferroptosis and investigate the effect of ACSL4 on nervous system diseases or injuries.

## 2. The Structure and Physiological Function of ACSL4

### 2.1. The Structure of ACSL4

ACSLs play a crucial role in activating long- and ultra-long-chain fatty acids to form fatty acid acyl-CoA esters. It is made up of five members of the ACSLs family and can be divided into two groups according to the composition of the homologous sequences. One is composed of ACSL1, ACSL5, and ACSL6, the other group consists of ACSL3 and ACSL4 [6]. The gene for *ACSL4* is situated on the X chromosome of the human body, and the subcellular localization of ACSL4 is mainly in the secretion pathway of endosome and peroxisome [7]. In addition, ACSL4 is transferred to the plasma membrane and the mitochondria-associated membrane, which is responsible for fatty acid synthesis and beta-oxidation [7]. ACSL4 is formed from five regions: an NH2 terminal, luciferase-like regions 1 and 2, the ligand that connects two luciferase-like regions, and a C terminal [8]. ACSL4 is highly expressed in the brain, adrenal glands, testes, and ovaries. In the ACSLs family, the luciferin-like region 2 and the C terminal amino acids sequence are identical, suggesting that the two regions are the crux of the reaction catalyzed by ACSLs. The absence of 50 NH2-corresponding amino acids may lead to differential responses of ACSLs to fatty acid preferences [6,9]. ACSL4 specifically exhibits preference for 20-carbon polyunsaturated fatty acid (PUFA) substrates, including arachidonic acid (AA) and adrenic acid (AdA) [10].

### 2.2. The Physiological Function of ACSL4

Long-chain fatty acids (carbon chain length 14 to 24) are significant nutrients for the formation and maintenance of cell membranes, energy supply with storage, membrane anchoring of proteins in protein post-translational modification (PTM) pathways, transport and localization pathways, and signal transduction, as well as protein interactions, etc. [6] First phase fatty acid transporters (FATPs) bind and transport long-chain fatty acids to target cells. ACSLs then catalyze the intracellular free long-chain fatty acids to acyl-CoA. PUFA is an acronym for polyunsaturated fatty acids, which are a category of fatty acids with multiple double bonds in their carbon chains. These fatty acids are divided into two primary groups: ω-3 (n-3) and ω-6 (n-6) fatty acids, based on the location of the first double bond from the methyl end of the fatty acid chain [11]. In the mammalian ACSLs family [12], ACSL4 tends to catalyze several PUFAs to polyunsaturated fatty acid coenzyme A (PUFAs-CoA), primarily including arachidonic acid 20:4 and adrenic acid 22:4. Following their formation, PUFAs-CoA are esterified into phospholipids by different lysophosphatidylcholine acyltransferases (LPCATs). This process facilitates the incorporation of long-chain polyunsaturated fatty acids into cellular lipid membranes [8]. It enhances membrane fluidity and facilitates the transportation of substances required for maintaining normal cellular physiological functions. ACSL4 is involved in distinct biochemical processes across different organelles. Within mitochondria, ACSL4 primarily contributes to fatty acid synthesis and β-oxidation [13]. Among the peroxidases, ACSL4 is mainly involved in β-oxidation and the synthesis of alkyl lipids. In the endoplasmic reticulum (ER), it promotes glycerolipid synthesis and ω-oxidation, serving as the primary pathway for catabolism of medium-chain fatty acids when β-oxidation is impaired [14,15]. The aforementioned findings highlight the critical involvement of ACSL4 in fatty acid metabolism, and it is the only subunit of the ACSLs family that plays an essential and direct role in the process of ferroptosis. However, the influence of ACSL4 on the catalytic selectivity for exogenous fatty acids can vary among different tissues and cell types, potentially owing to variations in cell type and intracellular fatty acid composition [16,17]. For example, the absence of ACSL4 in lipocytes reduces the incorporation of AA into phospholipids and correspondingly reduces the level of 4-hydroxynonenal, the lipid peroxidation product of AA [6]. ACSL4 deficiency plays a role in obesity-associated adipocyte dysfunction because the ability of PUFA to synthesize phospholipids is abruptly diminished, resulting in alterations in lipid composition and a high-fat diet and leading to fat accumulation and adipocyte death [18]. The overexpression of *ACSL4* in human arterial smooth muscle cells stimulates the production of phosphatidylinositol (PI) and phosphatidylinositol (PE) from exogenous AA, resulting in a decreased release of cytokine-dependent PGE2 [19,20]. Moreover, *ACSL4* expression stimulates the generation of PE from exogenous AA and oleic acid (OA) in fibroblast-like COS-7 cells. Dissociated AA is also implicated in the synthesis of phosphatidylcholine (PC) in COS-7 cells, and steroidogenic cells regulate AA release via the acyl-CoA thioesterase 2 (ACOT2) pathway [21]. In this pathway, ACSL4 catalyzes the conversion of free intracellular AA into AA-CoA and provides it to ACOT2, which subsequently transports AA to the mitochondria [22]. The released AA undergoes progressive conversion through the lipoxygenase pathway, leading to the formation of the steroidogenic acute regulatory (StAR) protein [17]. The StAR protein regulates the transport of cholesterol into the inner mitochondrial membrane and serves as a critical rate-limiting enzyme in steroid hormone biosynthesis [23]. AA and cyclic adenosine monophosphate (cAMP) transduce signals from hormone receptors into the nucleus through two different pathways and jointly regulate steroid production and STAR gene expression [6,24].

The overexpression of *ACSL4* may result in false positive results by disrupting protein distribution within cells. To investigate its physiological function, researchers commonly use gene silencing or knockout experiments. For example, knockout of *ACSL4* in rat fibroblast 3Y1 cells led to a reduction of AA-containing phospholipid levels following IL-1β stimulation, while AA-containing PC and PI levels were less affected [6,25]. In mouse embryonic fibroblasts, ACSL4 knockout significantly decreased the level of polyunsaturated fatty acid PE and inhibited ferroptosis [26]. Additionally, recombinant *glutathione peroxidase 4 (GPX4)-ACSL4* double knockout cells showed significant resistance to ferroptosis. ACSL4 facilitates the esterification of CoA to free fatty acids in an ATP-dependent manner, thereby activating fatty acid oxidation or lipid biosynthesis. This enzyme is also responsible for the enrichment of long-chain unsaturated ω-6 fatty acids in the cell membrane [27,28]. However, *ACSL4*-KO cells exhibited increased sensitivity to ferroptosis upon supplementation with exogenous AA or AdA (along with other long-chain PUFAs), likely due to the different kinetic properties of ACSL enzymes. ACSL4 prefers to use AA and AdA to synthesize phospholipids at low concentrations in the presence of other fatty acids, while other ACSL-like enzymes use other fatty acids. This concept is physiologically relevant because the molar percentage of plasma AA levels is at least one to two orders of magnitude lower than other fatty acids such as oleic acid, suggesting that ACSL4 is responsible for activating AA at physiological concentrations while other ACSL-like enzymes may activate AA when intracellular AA levels are elevated [26].

Thus, the regulation of PUFA by ACSL4 plays a crucial physiological role. Additionally, ACSL4 serves other physiological functions apart from its role in lipid metabolism. A study conducted by Cho revealed that *ACSL4*-deficient pure mice mostly perish during embryonic development, while heterozygous female mice with *ACSL4* deficiency experience decreased fertility and compromised offspring quality [29,30].

### 2.3. The Regulation Mechanism of ACSL4

*ACSL4* transcription is negatively regulated by miR-211-5p, miR-204A-5p, miR-34A-5p, miR-424-5p, miR-205, and miR-34a [5], the expression of which is also inhibited by the activation of integrinα6β4-mediated Src and signal transduction and transcription activator 3 (STAT3) or androgen receptors [31]. Furthermore, free AA may alter the levels of the ACSL4 enzyme through the promotion of ubiquitination and proenzyme degradation [32]. Moreover, cyclic adenosine monophosphate (cAMP), special protein 1 (SP1), tyrosine phosphatase SHP2 [33], and proto-oncogene transcriptional co-activator YAP were shown to positively regulate ACSL4 expression [5]. *ACSL4*’s proximal promoter region contains a cAMP response element binding site that initiates *ACSL4* transcription by binding to cAMP. The transcription of the *ACSL4* gene can be triggered by the release of YAP activity to enhance ferroptosis [34]. In addition, Zhang et al., found that the activation of PKCβII, one of the isoforms of PKC (protein kinase C), amplified lipid peroxidation through the phosphorylation and activation of ACSL4, which could directly phosphorylate ACSL4 Thr328. Furthermore, the lipid peroxidation PKCβII-ACSL4 positive feedback mechanism could enhance the level of lipid peroxidation to induce ferroptosis [35].

## 3. ACSL4 in Ferroptosis

As a form of cell death driven by iron-dependent lipid peroxidation, ferroptosis was proposed in 2012 by Drs. Brent R. Stockwell, Scott Dixon, and members of their laboratory [36,37,38]. In general, ferroptosis has three essential features: (1) oxidation of PUFAs (including membrane phospholipids); (2) redox activities related to iron utilization; (3) loss of lipid hydrogen peroxide (LOOH) repair capacity [8]. Morphologically, ferroptosis cells exhibit typical changes in mitochondrial membrane shrinkage, reduction or disappearance of mitochondrial cristae, and rupture of the outer membrane, whereas mitochondria usually show swelling in other forms of cell death [39,40,41]. Furthermore, treated with the ferroptosis inducer erastin, the nuclei of cancer cells retained their structural integrity and no nuclear pyknosis or chromatin edge clustering was observed [42]. These morphologic features distinguish ferroptosis from apoptosis and necrosis [43].

**Figure 1:**
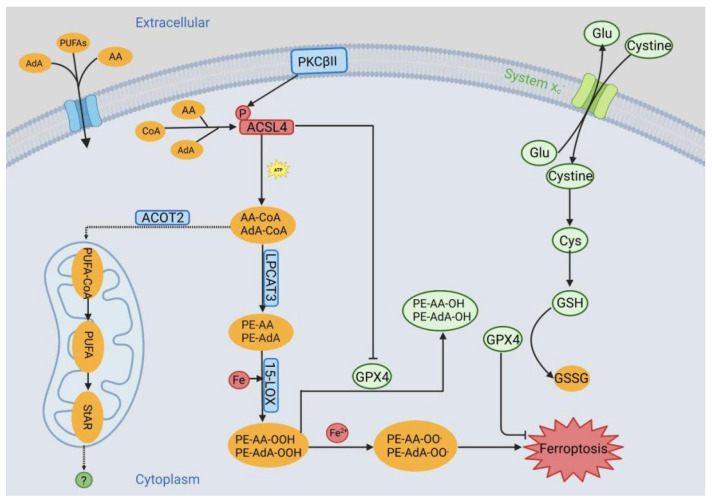
During ferroptosis, PKCβII phosphorylates the Thr328 site of ACSL4, directly activating ACSL4 and facilitating the biosynthesis of PUFA lipids. PUFAs, primarily AA 20:4 and AdA 22:4, are activated by ACSL4 and then form PUFA-CoA by binding with coenzyme A (CoA) at the endoplasmic reticulum oxidation center, a process that consumes adenosine triphosphate (ATP). PUFA-CoA is esterified to PUFA-PE through the assistance of LPCAT3. Subsequently, it undergoes oxidation by 15-lipoxygenase (15-LOX), resulting in the production of lipid hydroperoxides that contribute to iron depletion. Additionally, Fe^2+^ can be released from the labile iron pool, leading to the generation of reactive oxygen species (ROS) such as HO· through the Fenton reaction. Consequently, lipid peroxides, including LOOH, can accumulate via a similar reaction mediated by Fe, resulting in a chain reaction that produces a significant number of lipid radicals. System X_C_^−^ facilitates the exchange of cysteine and glutamate, enabling highly specific cysteine uptake. Once cysteine enters the cytoplasm, it undergoes reduction to cysteine, followed by catalysis by γ-glutamylcysteine synthase (γ-GCS) and glutathione synthase (GSS) to produce glutathione from cysteine. Two molecules of reduced glutathione (GSH) serve as electron donors, reducing PE-AA-OOH and PE-AdA-OOH to their respective alcohols, PE-AA-OH and PE-AdA-OH, and generating oxidized glutathione. Furthermore, ACSL4 directly inhibits GPX4, leading to ferroptosis. However, steroid-producing cells regulate the release of AA through the ACOT2 pathway. In this pathway, ACSL4 catalyzes the conversion of intracellular free AA to AA-coenzyme A and supplies it to ACOT2, which subsequently releases AA into the mitochondria. The released AA is progressively metabolized through the lipoxygenase pathway, inducing StAR, although its role in ferroptosis remains unclear. PUFAs, polyunsaturated fatty acids; AA, Arachidonic Acid; AdA, Adrenal Acid; PUFA-CoA, Polyunsaturated Fatty Acid-Coenzyme A; AA-CoA, Arachidonic Acid-Coenzyme A; AdA-CoA, Adrenal Acid-Coenzyme A; ATP, Adenosine Triphosphate; LPCAT3, Lysophosphatidylcholine acyltransferase 3; PE-AA, Phosphatidylethanolamine-Arachidonic Acid; PE-AA, Phosphatidylethanolamine-Adrenal Acid; 15-LOX, 15-lipoxygenase; PE-AA-OOH, Phosphatidylethanolamine-Arachidonic Acid Hydroperoxide; PE-AdA-OOH, Phosphatidylethanolamine-Adrenal Acid Hydroperoxide; PE-AA-OO**^.^**, Phosphatidylethanolamine-Arachidonic Acid Peroxyl Radical; PE-AdA-OO**^.^**, Phosphatidylethanolamine-Adrenal Acid Peroxyl Radical; PE-AA-OH, Phosphatidylethanolamine-Arachidonic Acid Alcohol; PE-AdA-OH, Phosphatidylethanolamine-Adrenal Acid Alcohol; Glu, Glutamic Acid; Cys, Cysteine; GSH, Glutathione; GSSG, Glutathione Disulfide; GPX4, Glutathione Peroxidase 4; ACOT2, acyl-CoA thioesterase 2; StAR, steroidogenic acute regulatory; PKCβII, protein kinase C βII.

Alterations in fatty acid metabolism serve as markers that indicate various pathological conditions and metabolic disorders. Lipid metabolism disorders are observed in ferroptosis and are heavily reliant on specific lipid metabolism proteins involved in the metabolism of AA and AdA. To identify the major genes associated with lipid peroxidation in ferroptosis, the research teams of Sebastian Doll and Bettina Proneth performed two independent genetic experiments. By simultaneously analyzing a short palindromic repeat-based genetic screen and another transcriptome microarray assay after comparing ferroptosis-sensitive and resistant cells, *ACSL4* gene expression was found to be indispensable in the process of the oxidation of arachidonic acid-phosphatidylethanolamine (AA-PE) and adrenal acid-phosphatidylethanolamine (AdA-PE) [26]. Among the ACSLs family members, ACSL4 is the lipid metabolism enzyme which is most closely related to ferroptosis. The overexpression of *ACSL4* leads to the catalysis of diverse PUFAs, including AA/AdA, thereby modifying the composition of cellular lipids and increasing cellular susceptibility to ferroptosis [44]. In general, AdA and AA are first activated by ACSL4 during ferroptosis, followed by the formation of AdA-CoA and AA-CoA derivatives at ER-associated oxidation centers. AdA-CoA and AA-CoA are then esterified by LPCAT3 to AdA-PE and AA-PE, which are then oxidized by 15-lipoxygenase (15-LOX) to produce lipid hydroperoxide, giving rise to ferroptosis (Figure 1).

By cloning the mouse *ACSL4* gene cDNA into a lentiviral vector, Yu Cui and Yan Zhang demonstrated that cortical lentivirus administration injected into the left brain after tMCAO surgery resulted in increased infarct size and decreased neurological function in the *ACSL4*-overexpressing brain. Additionally, confocal microscopy revealed neuronal death and heightened microglial activation in *ACSL4*-overexpressing mice, leading to the release of substantial amounts of neurotoxic factors such as reactive oxygen species (ROS) [45,46]. In the cerebral ischemia/reperfusion model, *ACSL4* knockdown attenuates ischemic brain injury while *ACSL4* overexpression exacerbates ischemic brain injury [26]. Furthermore, ACSL4 contributes to neuronal death by promoting ferroptosis, and therefore, inhibiting the esterification of AA/AdA to PE through pharmacological or genetic inhibition of *ACSL4* has emerged as a specific approach to counteracting ferroptosis [47]. ACSL4 is also a potential target for tumor treatment, as studies have demonstrated its inhibitory effect on glioma cell proliferation through the activation of the ferroptosis pathway [48]. Suppression of the thrombin-ACSL4 pathway may reduce neuronal ferroptosis following ischemic stroke [49]. Additionally, paeonol exhibits significant inhibition of ACSL4-mediated neuronal ferroptosis induced by ferroptosis inducers [50]. In conclusion, ACSL4-mediated fatty acid activation of AA/AdA is a key step in ferroptosis. The expression level or enzyme activity of the ACSL4 protein is a vital biological factor for ferroptosis in cells and tissues, which can be used as a biomarker for ferroptosis susceptibility and as a therapeutic target for the treatment of ferroptosis-related diseases. Overall, the overexpression of *ACSL4* promotes ferroptosis by regulating PUFAs, particularly when PUFAs reach hazardous levels.

Recently, Leslie Magtanong et al., discovered that ACSL4 serves as a context-specific regulator of ferroptosis. Through an overview of previous studies, Magtanong highlighted ACSL4’s role in inducing ferroptosis, primarily attributed to its inhibitory effect on GPX4 [51]. The relationship between ACSL4 and GPX4 has been a prominent area of investigation in ferroptosis research. For instance, the team led by Bo Chu demonstrated that ACSL4 is necessary in ferroptosis induced by erastin or GPX4 inhibitors, whereas it is dispensable in P53-mediated ferroptosis [52]. Shui et al., also reported that lipids can be directly generated through photodynamic therapy (PDT) with exogenous oxygen radicals, initiating lipid peroxidation independent of ACSL4 and lipoxygenases (ALOXs) [53]. Furthermore, Pang et al., identified that edaravone can alleviate spinal cord injury by modulating the GPX4/ACSL4/5-LOX pathway [54]. Li et al., discovered that baicalein improves cerebral ischemia-reperfusion injury through the GPX4/ACSL4/ACSL3 axis [55]. Wang et al., demonstrated that Seco Lupan Triterpen Derivatives induce ferroptosis via the GPX4/ACSL4 axis [56]. These findings provide a theoretical foundation for further elucidating the mechanisms of ferroptosis.

## 4. ACSL4 in Neurological Diseases and Injuries

### 4.1. ACSL4 in Brain Injury

Traumatic Brain Injury (TBI) is one of the world’s most serious health problems with high morbidity and mortality [57,58]. TBI and its complications place an enormous economic burden on families and society [59,60,61,62], and an increasing number of studies have shown that ACSL4 plays an important role in the process of ferroptosis induced after TBI [63]. ACSL4 turns membrane phospholipids into AA/AdA-CoA, which is the initial step to lipid peroxides [64]. Hogan discovered the elevated level of PUFAs in TBI, and that the occurrence of lipid peroxidation-mediated injury is associated with brain injury [65]. Xiao found that, 6 h after controlled cortical injury (CCI), the mRNA level of *ACSL4* increased [66], and a significant increase in ACSL4 was observed after injury according to Kenny [67] (Table 1). However, as biomarkers related to ferroptosis, GPX4 and ACSL4 [68,69] were differentially expressed only in the early post-TBI period, suggesting that the most active stage of ferroptosis may occur early after injury. Using baicalein could abate PE oxidation and provided histological and cognitive protection in postinjury [67,70,71] (Table 1). Furthermore, the application of ferroptosis inhibitors ferristatin-1 and ferristatin II in the TBI mouse model can inhibit iron deposition, neuronal degeneration, and reduce brain injury of TBI [72], which testifies the existence of ferroptosis in TBI. As previously discussed, the accumulation of oxidized AA- or AdA-containing PE leads to ferroptosis. Therefore, inhibition of ACSL4 and thus the formation of AA- and AdA-esterified PE may also protect against TBI.

Moreover, knockdown of *ACSL4* by specific shRNA inhibited erastin-induced ferroptosis in HepG2 and HL60 cells (ferroptosis-sensitive cells) [69]. The inhibition of *ACSL4* expression by shRNA only reduced MDA production, thus reducing the final production of lipid peroxidation, while Fe^2+^ did not accumulate in HepG2 and HL60 cells after erastin treatment. These findings suggest that ACSL4 induces neuronal ferroptosis by regulating lipid peroxidation rather than iron accumulation.

### 4.2. ACSL4 in Stroke

#### 4.2.1. ACSL4 in Ischemic Stroke

Ischemic stroke is currently one of the leading causes of human mortality, accounting for 80% of all strokes [79]. Currently, the only treatment options for patients with ischemic stroke are surgery or thrombolysis with tissue plasminogen activator, but the prognosis remains poor [80]. Recent studies have found that ferroptosis is closely related to the onset and development of stroke, which may be a potential direction for stroke treatment [81].

The ferroptosis inhibitors liproxstatin-1 and ferrostatin-1 could prevent cerebral ischemia reperfusion injury induced by stroke in mice [82]. To observe the temporal pattern of *ACSL4* expression after focal ischemia, Cui et al., subjected mice to transient middle cerebral artery occlusion (tMCAO) for 1 h followed by reperfusion. The expression of *ACSL4* in the ipsilateral cortex decreased significantly after 1 to 3 h of ischemia and was higher than that in the contralateral cortex after 6 h of reoxygenation. This suggests that, in the early stages of focal ischemia, the expression of *ACSL4* is down-regulated [46]. Hypoxia-inducing factor 1-alpha (HIF-1α) mediated decreased *ACSL4* expression after oxygen and glucose deprivation (OGD). Knockdown of *ACSL4* can alleviate ischemic brain damage, and the overexpression of *ACSL4* can exacerbate ischemic brain damage [83,84]. Chen et al., established a transient ischemic model in mice with middle cerebral artery occlusion (MCAO) after intravenous administration of rosiglitazone 1 h before MCAO. After inhibition of ACSL4 with rosiglitazone (RSG), the decrease of GPX4 was greatly attenuated (Table 1). Neurological function was significantly improved at 72 h after stroke, and cerebral infarct volume was reduced. This study demonstrated that inhibition of ACSL4 could promote recovery of neurological function after stroke by inhibiting ferroptosis [73]. Tuo et al., found that, during the period of I/R, reduction of ACSL4 could be the result of modification after translation. They also discovered that ACSL4 can mediate thrombin cytotoxicity which can be blocked by the ACSL4 inhibitor pioglitazone (PIO). These results suggest that thrombin may contribute to neuronal cell death through the promotion of ACSL4-dependent ferroptosis, and that reduction of ACSL4 may contribute to the inhibition of thrombin-induced ferroptosis [49].

#### 4.2.2. ACSL4 in Hemorrhagic Stroke

Intracerebral hemorrhage (ICH) is one of the most common and refractory diseases in the world [85]. The hematoma after intracerebral hemorrhage causes progressive brain tissue damage [86], and it is closely associated with ferroptosis during its development [87]. *ACSL4* mRNA expression was significantly increased in brain microvascular endothelial cells (BMVECs) treated with the hypothermic oxygen–glucose deprivation intracerebral hemorrhage model cells (OGD/H). ACSL4 inhibits miR-106b-5p, promoting ferroptosis. Target gene analysis identified *ACSL4* as a target gene of miR-106b-5p in OGD/H ICH model cells [46] (Table 1). The overexpression of *ACSL4* countered the effects of miR-106b-5p, suppressed the viability of ICH cells, and stimulated ferroptosis. These results suggest that ACSL4 promotes ferroptosis, decreasing the cellular function of BMVECs, which is consistent with the findings of Xie et al. [88]. Furthermore, H19 acts as a competing endogenous RNA ceRNA and regulates the proliferation and ferroptosis of BMVECs through the miR-106b-5p/ACSL4 axis [81,82]. *H19* knockdown may prevent ICH by regulating miR-106b-5p/ACSL4, making this axis a potential therapeutic target for ICH treatment.

Paeonol (PAN, 2′-hydroxy-4′-methoxy acetophenone) is a natural product isolated from Paeoniflora [89]. Zheng’s team used hemin to mimic ICH in HT22 cells and found that hemin significantly up-regulated *ACSL4* expression in neuronal cells, while PAN partially reversed this phenomenon. Additionally, RNA pull-down experiments identified *UPF1* and *ACSL4* as downstream targets of HOTAIR in ICH, and PAN could inhibit ICH progression by mediating the HOTAIR/UPF1/ACSL4 axis, which may serve as a new medicine for cerebral hemorrhage [50,89] (Table 1).

In recent years, the role of ferroptosis in early brain injury (EBI) of subarachnoid hemorrhage (SAH) has been highlighted. Ferroptosis is involved in the pathogenesis of EBI after SAH through various pathways, including the activation of ACSL4, iron metabolism disorders [90], and the down-regulation of *GPX4* and ferroptosis suppressor protein 1 (FSP1) [91]. Western blot and immunofluorescence experiments have confirmed the expression level of ACSL4 in brain tissue after SAH, which increases and then decreases. The immunofluorescence assay also revealed the colocalization of ACSL4 with the neuronal marker NEUN in the brain, which significantly increased 24 h after SAH [92]. Using siRNA technology to silence *ACSL4* expression, inflammation, blood–brain barrier (BBB) damage, oxidative stress, cerebral edema, behavioral and cognitive deficits, and neuronal death were reduced, while the number of surviving neurons increased. Similar results were obtained with ferroptosis inhibitors [27]. Therefore, early intervention to reduce the oxidative response and ferroptosis may be an effective treatment for SAH. For example, puerarin can activate SIRT1 or the AMPK/PGC1α/NRF2 pathway to alleviate oxidative stress and reduce ferroptosis in EBI after SAH [93].

### 4.3. ACSL4 in Alzheimer’s Disease

Alzheimer’s disease (AD) is a degenerative disease of the central nervous system that primarily affects people over the age of 65 [94]. Its clinical manifestations are mainly the decline of memory, language, and other cognitive abilities. The main pathological features are amyloid beta peptide (Aβ) [95,96] as the core component of senile plaques and neurofibrillary tangles caused by tau hyperphosphorylation [97,98]. Under the increasing aging trend, due to the unclear pathogenesis of Alzheimer’s disease and the lack of cost-effective clinical treatment, Alzheimer’s disease places a heavy burden on patients and medical social security [97,99].

Lipids constitute a vital component of the brain, comprising approximately 40% to 75% of its dry weight and accounting for up to 80% of the myelin sheath. They play crucial roles in energy metabolism, signal transduction, and various other processes [100]. In the context of AD, the elevated presence of free radicals leads to lipid peroxidation, which is closely associated with the initial pathological changes observed in AD [101]. Furthermore, there is evidence linking ROS to brain damage in AD [102]. Previous studies have shown that the level of total free fatty acids in the hippocampus of AD patients is significantly decreased and the level of ACSL4 is significantly increased. Furthermore, high levels of free MDA and 4-hydroxynonenal (4-HNE) are detected in several brain regions, and *GPX4* expression is down-regulated, proving the existence of ferroptosis and lipid peroxidation in AD brain [103]. The abnormal folding and aggregation of Aβ in the brain is one of the hallmark pathological changes of Alzheimer’s disease [104]. It has been reported that Aβ oligomer can cause long-term enhancement impairment in the hippocampus of experimental rats, and abnormally activate microglia proinflammatory phenotype and complement system, inducing neuroinflammation and synaptic loss. Aβ is potentially associated with lipid peroxidation in ferroptosis [104], it has the ability to integrate into the lipid bilayer of neurons, leading to the production of hydrogen peroxide. However, in the presence of oxidation-reducing metal ions, such as Fe^2+^, a Fenton reaction can occur, resulting in the generation of a substantial amount of ROS that further target unsaturated lipids. This exacerbates oxidative damage to lipids, proteins, and DNA [105]. Praticò et al., observed the accumulation of Aβ through lipid peroxidation and oxidative stress in an APP mouse model [74]. Gao et al., conducted experiments using tetrahydroxy stilbene glycoside (TSG) on mouse models of AD and found that TSG reduced the formation and accumulation of Aβ (Table 1). Furthermore, compared to the non-intervention group, the TSG-treated group exhibited a certain reduction in indices associated with lipid peroxidation in ferroptosis [75].

### 4.4. ACSL4 in Parkinson’s Disease

Parkinson’s disease (PD) is a widespread chronic degenerative disorder that commonly affects motor skills, language, and other functions of the central nervous system [106,107]. PD is characterized by the damage to dopamine (DA) neurons in the pars compactus nigra (SNpc), which may result in muscle rigidity, static tremors, sleep disturbances, motor retardation, abnormal postural reflexes, sensory disturbances, autonomic nervous system dysfunction, and other clinical symptoms [76]. 

Through the assessment of PD patients, it has been observed that the iron content in glial and dopaminergic neurons is abnormally elevated compared to that in healthy individuals, and this elevation is positively associated with the severity of PD [108,109,110]. Research has identified the formation of Lewy bodies, composed mainly of α-synuclein nucleoprotein [111,112], as a distinctive hallmark of PD, occurring within the cytoplasm of substantia nigra neurons [113,114]. Notably, α-synuclein exhibits a strong affinity for lipid binding. In addition, α-synuclein not only mediates the formation of membrane PUFAs [115], but also regulate the metabolism of AA [77]. ACSL4 demonstrates a specific preference for AA, and PUFA has the ability to induce α-synuclein aggregation [116,117].

Recent research has indicated that the inhibition of SP1 can confer neuroprotective effects in PD models [118]. Additionally, Ma et al., demonstrated that SP1 has the ability to reverse the impact of repressor element-1 silencing transcription factor (REST) on erastin-induced LUHMES cell viability, ROS production, ferroptosis, and neuronal damage, implying that REST may alleviate PD by reducing SP1 activity [118]. Additionally, the study revealed that the overexpression of *REST* down-regulates *ACSL4* in erastin-induced LUHMES cells [118]. The interaction between miR-494-3p and *ACSL4, REST, or SP1* was examined using luciferin chromatin immunoprecipitation or EMSA. The results revealed that the repression of miR-494-3p could prevent ferroptosis and neuronal damage by regulating the *SP1/ACSL4* axis in PD by targeting *REST*. *REST* is a downstream gene of miR-494-3p, which can inhibit ferroptosis neuronal damage induced by SP1, ROS, and mitochondrial damage in LUHMES cells [118]. Furthermore, Song et al., discovered that 1-methyl-4-phenyl-1,2,3,6-tetrahydropyridine (MPTP) treatment significantly up-regulates ACSL4 expression and down-regulates GPX4 expression in PD mice (Table 1). Apoferritin [119] treatment leads to reduced ASCL4 expression and increased expression of ferroptosis suppressor protein 1 (FSP1) [76]. Moreover, Yu et al., demonstrated that β-hydroxybutyrate (BHB) directly affects the stability of *ACSL4* mRNA through zinc finger protein 36 (ZFP36), exerting inhibitory effects on ferroptosis [77] (Table 1). The aforementioned studies offer insights for potential future PD treatments by targeting ACSL4 to inhibit ferroptosis [109].

### 4.5. ACSL4 in Spinal Cord Diseases

Spinal cord injury causes permanent or temporary changes in the function of the spinal cord which can be divided into traumatic spinal cord injury (TSCI) and nontraumatic spinal cord injury (NTSCI) [120]. The main symptoms include sensory–motor or autonomic nerve dysfunction below the level of the spinal cord injury. TSCI is usually caused by external physical shocks, such as car accidents, falls, sports, falling objects, or violent activities. Globally, with the popularity of modern cars, the rise of various outdoor sports, and the growth of the aging population, the incidence of TSCI presents an increasing and aging trend.

Amyotrophic lateral sclerosis (ALS) is a neurodegenerative disorder characterized by the progressive degeneration of motor neurons in the central nervous system, including the brain and spinal cord. This degeneration leads to muscle paralysis, atrophy, and functional impairment [121]. Although ALS and TSCI are distinct diseases, they share certain similarities [122]. In rare cases, ALS can cause spinal cord injury, while TSCI can result in motor neuron injury resembling ALS. Furthermore, both conditions are associated with neurological damage and dysfunction, significantly impacting the affected individuals’ lives [123]. Edaravone shows promise as a potential therapeutic intervention by preventing ferroptosis in ALS [124].

Edaravone, also known as 3-methyl-1-phenyl-2-pyrazolin-5-one, is a free radical scavenger due to the lipophilicity of phenylmethyl, which allows edaravone to remain on the membrane and scavenge lipid-reactive oxygen species [125,126]. By scavenging free radicals, edaravone has the potential to mitigate oxidative stress and inhibit the activation of ACSL4, and Yilin Pang and colleagues demonstrated that edaravone inhibits the ferroptosis pathway following spinal cord injury in a contusion injury model [54]. 5-LOX and ACSL4 increased 2 days after injury, while edaravone significantly down-regulated their expression and up-regulated *GPX4/xCT* in the acute phase of spinal cord injury [54]. There was no significant change in the expression of 5-LOX and ACSL4 in each group 7 days after SCI, suggesting that ferroptosis mainly occurred in the acute phase [54]. Edaravone regulates GPX4/ ACSL4/5-LOX in the lower spinal segment of the lesion, and ACSL4 is expressed in both the nucleus and cytoplasm of the injured spinal cord [54] (Table 1).

In addition, abnormalities in mitochondrial function and morphology have been observed in ALS. In the context of neurodegenerative diseases, edaravone has demonstrated a protective effect on mitochondria [127]. However, the precise mechanism underlying this effect remains unclear. Considering that ACSL4 is localized to mitochondria-associated membranes (MAMs), it is plausible that edaravone’s action on mitochondrial integrity may prevent ACSL4 dysregulation or degradation [128]. Additionally, edaravone has been shown to possess anti-inflammatory properties by inhibiting the production of inflammatory mediators. By attenuating the inflammatory response, edaravone may indirectly modulate ACSL4 levels and potentially impact the pathogenesis of TSCI [129].

### 4.6. ACSL4 in Multiple Sclerosis

Multiple sclerosis (MS) is a disease characterized by inflammatory demyelination, which involves the infiltration of immune cells into the central nervous system (CNS) [130,131], leading to recurrent demyelinating lesions with varying degrees of inflammation, including inflammation throughout the entire lesion area, limited to the lesion border or lack, and all of these were observed in MS patients [132]. Moreover, MS may cause ongoing neurodegeneration (secondary progression), which leads to cumulative disability over time [133]. To date, treatment of MS has reduced the frequency of relapses without affecting secondary progression. Iron acts as a co-factor for several enzymes that maintain oligodendrocyte and myelin health, and may play a crucial role in remyelination [134]. Aberrant iron regulation in multiple sclerosis (MS) has been observed through magnetic resonance imaging (MRI) and histological examinations, revealing iron deposition in gray matter and a decrease in normal white matter [132]. These findings suggest a potential association between MS and ferroptosis. 

Given that MS is an immune-mediated disease with similarities to autoimmune disorders, several drugs for MS have been developed using the experimental autoimmune encephalitis (EAE) model. Genes implicated in ferroptosis were examined in the spinal cord of EAE mice. The results indicated significant alterations in key ferroptosis-related genes, including *ACSL4* and *GPX4*. Furthermore, elevated levels of ACSL4 were detected prior to the onset of clinical symptoms in EAE mice, and remained high in the chronic active areas of MS patients. Notably, during the peak of EAE, the expression of *ACSL4* was significantly increased [135]. Knocking down the *ACSL4* gene considerably reduced the severity of EAE and the clinical score of EAE mice, indicating that ACSL4-mediated ferroptosis provoked inflammation and promoted T-cell activation and CNS infiltration (Table 1). Therefore, the inhibition of *ACSL4* suppresses ferroptosis, which provides a potential therapeutic target for the treatment of secondary neurodegeneration, but further clinical trials are needed to test the efficacy of the drug [78,136].

## 5. Conclusions

Ferroptosis is an iron-dependent and novel form of regulated cell death in which lipid peroxide levels accumulate to lethal levels. A variety of diseases, including nervous system disorders and injuries, are associated with ferroptosis. According to reports on ferroptosis, ACSL4 is one of the important enzymes in the ferroptosis pathway, which can be used as a biomarker for ferroptosis and can promote ferroptosis. On the one hand, it can promote tumor cell death; on the other hand, it also illustrates its role in disease-induced ferroptosis. The pro-ferroptosis effect of ACSL4 was mainly due to its critical role in AA and AdA metabolism and lipid peroxidation. Due to the different content and distribution of fatty acids in different tumor cells and different tissue cells, as well as the different distribution and content of ACSL4 and other related lipid metabolism enzymes, the sensitivity of different tissues and different cells to ferroptosis varies greatly [135,137]. For example, *ACSL4* is highly expressed in patients with liver cancer and colon cancer, and the higher the expression level is, the worse the prognosis is, while patients with gastric cancer [138] tend to have a low expression of *ACSL4*. This is a very important clue for how to use ferroptosis to inhibit tumor cells or inhibit ferroptosis to improve the prognosis of neurological diseases. At present, it is urgent to accelerate the process of ferroptosis treatment of tumors without toxic side effects on normal tissues through the pathological tissue analysis of tumors, or by further exploring the time window of ACSL4 inhibitors or other ferroptosis inhibitors after ischemia-reperfusion injury [139,140,141]. In addition, the possibility that ACSL4 plays a role in genetic disorders cannot be ignored, as ACSL4 deletion mutations have been reported in a family with Alport disease (also known as eye-ear-kidney syndrome). Moreover, the specifics of ACSL4 deletion and complex disorders remain to be investigated. Finally, there are still few studies on the lipid peroxidation pathway associated with ACSL4 in TBI. As a type of injury with a high mortality rate, the role of ACSL4 in TBI needs to be further and more widely explored.

## Figures and Tables

**Table 1:** Models of neurodegenerative diseases and outcomes after intervention.

Diseases	Biological Model	Intervention Measure	Consequence	Reference
Traumatic Brain Injury	Controlled cortical impact (CCI)	/	ACSL4 expression level increased	[66]
	CCI	Baicalein	Decreased ferroptotic PE oxidation	[67]
Ischemic stroke	Middle cerebral artery occlusion (MCAO)	/	ACSL4 increased after decreasing 1–3 h of ischemia	[46]
	MCAO	liproxstatin-1/Rosiglitazo-ne/	Lipid peroxidation index was significantly inhibited in comparison with untreated group	[73]
Hemorrhagic stroke	Oxygen and glucose deprivation (OGD)	/	*ACSL4* mRNA expression was significantly increased	[46]
	OGD	Paeonol	Paeonol inhibited the expression of ACSL4	[50]
Alzheimer’s disease	APPswe transgenic mice	/	Aβ accumulates in brain tissue due to lipid peroxidation	[74]
	APPswe/PSEN1dE9 (APP/PS1) double transgene mice	tetrahydroxy stilbene glycoside (TSG)	TSG inhibited the expression of ACSL4	[75]
Parkinson’s disease	PD mice model	1-methyl-4-phenyl-1,2,3,6-tetrahydropyridine (MPTP)	The expression of ACSL4 significantly increased	[76]
	PD mice model	β-hydroxybutyrate (BHB)	BHB inhibits ferroptosis in PD model	[77]
Spinal cord injury	Spinal cord	Edaravone	Reduces ACSL4 levels	[54]
	contusion injury model			
Multiple sclerosis	Experimental autoimmune encephalitis (EAE) model	*ACSL4*-KO	Knocking down the *ACSL4* gene considerably reduced the severity of EAE and the clinical score of EAE mice	[78]

## Data Availability

Data sharing is not applicable to this article as no datasets were generated or analyzed during the current study.
